# The Inhibitory Effects of Cobalt Protoporphyrin IX and Cannabinoid 2 Receptor Agonists in Type 2 Diabetic Mice

**DOI:** 10.3390/ijms18112268

**Published:** 2017-10-28

**Authors:** Christina McDonnell, Sergi Leánez, Olga Pol

**Affiliations:** 1 Grup de Neurofarmacologia Molecular, Institut d’Investigació Biomèdica Sant Pau, 08025 Barcelona, Spain; christina.mcdonnell@e-campus.uab.cat (C.M.); sergi.leanez@uab.es (S.L.); 2 Institut de Neurociències, Universitat Autònoma de Barcelona, 08193 Barcelona, Spain

**Keywords:** antinociception, cannabinoid receptors, diabetic neuropathy, heme oxygenase 1, NAD(P)H: quinone oxidoreductase 1, Nrf2 transcription factor

## Abstract

The activation of the transcription factor Nrf2 inhibits neuropathy and modulates the activity of delta-opioid receptors (DOR) in type 2 diabetic mice but the impact of Nrf2/HO-1 pathway on the antinociceptive actions of cannabinoid 2 receptors (CB2R) has not been assessed. Using male mice BKS.Cg-m+/+Leprdb/J (db/db) we investigated if treatment with cobalt protoporphyrin IX (CoPP), an HO-1 inductor, inhibited mechanical allodynia, hyperglycemia and obesity associated to type 2 diabetes. The antinociceptive effects of JWH-015 and JWH-133 (CB2R agonists) administered with and without CoPP or sulforaphane (SFN), a Nrf2 transcription factor activator, have been also evaluated. The expression of Nrf2, HO-1, NAD(P)H: quinone oxidoreductase 1 (NQO1) and c-Jun N-terminal kinase (JNK) in sciatic nerve and that of the CB2R on the dorsal root ganglia from animals treated with CoPP and/or SFN were assessed. CoPP treatment inhibited allodynia, hyperglycemia and body weight gain in db/db mice by enhancing HO-1/NQO1 levels and reducing JNK phosphorylation. Both CoPP and SFN improved the antiallodynic effects of JWH-015 and JWH-133 and expression of CB2R in db/db mice. Therefore, we concluded that the activation of antioxidant Nrf2/HO-1 pathway potentiate the effects of CB2R agonists and might be suitable for the treatment of painful neuropathy linked to type 2 diabetes.

## 1. Introduction

Diabetic neuropathy is a serious disorder associated with diabetes, which diminishes the quality of life [1]. It is often described as a tingling or burning sensation that is accompanied by allodynia and hyperalgesia [2]. However, and despite being an important problem for diabetic patients, current treatments are not fully effective [3].

Oxidative stress and inflammation caused by hyperglycemia played a prominent role in the pathogenesis of diabetes. Both are involved in the neurochemical, metabolic, and vascular alterations on the peripheral nerves occasioning damages and consequent peripheral neuropathy in diabetic patients [2,4]. Interestedly, the enzyme heme oxygenase 1 (HO-1) participates in the mitigation of deleterious effects generated by oxidative stress [5]. That is, the overexpression of HO-1 diminishes oxidative stress and inflammatory responses activated by hyperglycemia [6]. The activation of HO-1 also caused powerful antinociceptive effects during acute, inflammatory, visceral and neuropathic pain [7,8,9,10], as well as in neuropathy related to type 1 diabetes [11], although the influence of this enzyme on the inhibition of allodynia linked to type 2 diabetes has not evaluated.

Furthermore, another important antioxidant enzyme activated by the transcription factor Nrf2 is the NAD(P)H quinone oxidoreductase 1 (NQO1). The up-regulation of this enzyme modulates oxidative stress and abundant neuro-inflammatory responses also accountable for diabetic neuropathy induction [12,13]. Nevertheless, the effects of CoPP (an HO-1 inducer compound) on the expression of this enzyme in type 2 diabetic animals have not been assessed.

Numerous studies demonstrated that mitogen activated protein kinases (MAPK) participate in the regulation of several chronic diseases including diabetes [14]. That is, the extracellular signal-regulated protein kinase (ERK1/2) and p38 contribute to the pain sensitivity observed in the first stages of type 2 diabetes [15,16]. Increasing evidences also proved that c-Jun N-terminal kinase (JNK) regulates inflammatory and neural plasticity responses caused by nerve injury and type 1 diabetes leading to pain hypersensitivity [17,18]. Moreover, a remarkable activation of JNK in the sciatic nerve from db/db or ob/ob mice contributing to pain sensitivity and insulin resistance was established [19,20]. Nevertheless, the effect of treatment with CoPP on the modulation of JNK activation in db/db mice remains unidentified.

The inhibitory role-played by cannabinoid 2 receptors (CB2R) in neuropathic pain are well documented. A recent investigation demonstrated that the administration of a CB2R agonist (JWH-015) dose dependently inhibited allodynia in 1 diabetic mice [21]. In accordance, other findings also shown that the administration of non-selective CB1R/CB2R agonists (WIN 55,212-2) inhibited painful neuropathy in animals with type 1 or type 2 diabetes [22,23]. In this study, we examined the antinociceptive actions of two selective CB2R agonists in animals with type 2 diabetes.

Moreover, given that oxidative stress induced by hyperglycemia is a key factor for the progress of diabetic neuropathy and that HO-1 triggered by the transcription factor Nrf2 potentiated the effects of delta-opioid receptors (DOR) in type 2 diabetic mice [20], we hypothesized that pre-treatment with CoPP or sulforaphane (SFN), a Nrf2 transcription factor activator, might increase the analgesic efficacy produced by selective CB2R agonists in animals with type 2 diabetes.

Therefore, in BKS.Cg-m+/+Leprdb/J (db/db) male mice which develop type 2 diabetes, with mechanical allodynia, hyperglycemia and obesity from 6-12 weeks of age [20,24,25], we investigated whether treatment with CoPP attenuated them through regulating the Nrf2, HO-1, NQO1 and JNK protein levels in the sciatic nerve of these animals. The contribution of CoPP and SFN on the antiallodynic actions and expression of CB2R in the dorsal root ganglia from db/db mice were also analyzed.

## 2. Results

### 2.1. Diabetic Neuropathy

Our results indicated that glucose levels from db/db mice (484.8 ± 10.2 mg/dL) were higher than in db/+ mice (145.3 ± 11.1 mg/dL) (*p* < 0.001; Student’s *t*-test). Moreover, an increased body weight was demonstrated in db/db mice (39.6 ± 0.8 g vs. 21.7 ± 0.3 g in db/+ mice; *p* < 0.01; Student’s *t*-test). Mechanical allodynia was also confirmed in the von Frey filaments (1.6 ± 0.1 von Frey filament strength, g) in db/db as compared to db/+ mice (2.7 ± 0.1 (von Frey filament strength, g)) (*p* < 0.001; Student’s *t-*test).

### 2.2. Effects of CoPP on Mechanical Allodynia

**Figure 1:**
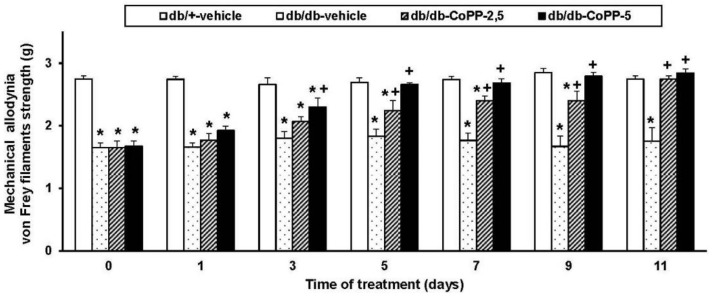
The inhibition of mechanical allodynia induced by CoPP in db/db mice. Mechanical allodynia in the hind paws of db/db mice intraperitoneally treated with vehicle or CoPP (2.5 and 5 mg/kg) during 11 consecutive days is shown. The effects of vehicle in db/+ mice are also represented. Data are shown at day 0, 1, 3, 5, 7, 9 and 11 of CoPP treatment and expressed as von Frey filaments strength (g). For each day evaluated, * indicates significant differences vs. db/+ mice treated with vehicle and + indicates significant differences vs. db/db mice treated with vehicle (*p <* 0.05, one-way ANOVA followed by the Student Newman Keuls test). Each column represents the mean and vertical bars indicate the standard error of the mean (SEM); *n* = 6 animals for each group.

The antiallodynic effects of 2.5 and 5 mg/kg of CoPP intraperitoneally administered were evaluated at days 0, 1, 3, 5, 7, 9 and 11 of treatment (Figure 1). Two way ANOVA repeated measures indicated significant effects of the treatment (*p <* 0.001), time of administration (*p* < 0.001) and its interaction (*p* < 0.001). Indeed, while mechanical allodynia was completely reversed at 5 days of treatment with 5 mg/kg of CoPP (*p* < 0.001; one-way ANOVA vs. vehicle db/db treated mice), 11 days of continuous treatment with 2.5 mg/kg of CoPP are needed (*p* < 0.001; one-way ANOVA vs. vehicle db/db treated mice).

### 2.3. Effects of CoPP on Blood Glucose Levels and Body Weight

**Figure 2:**
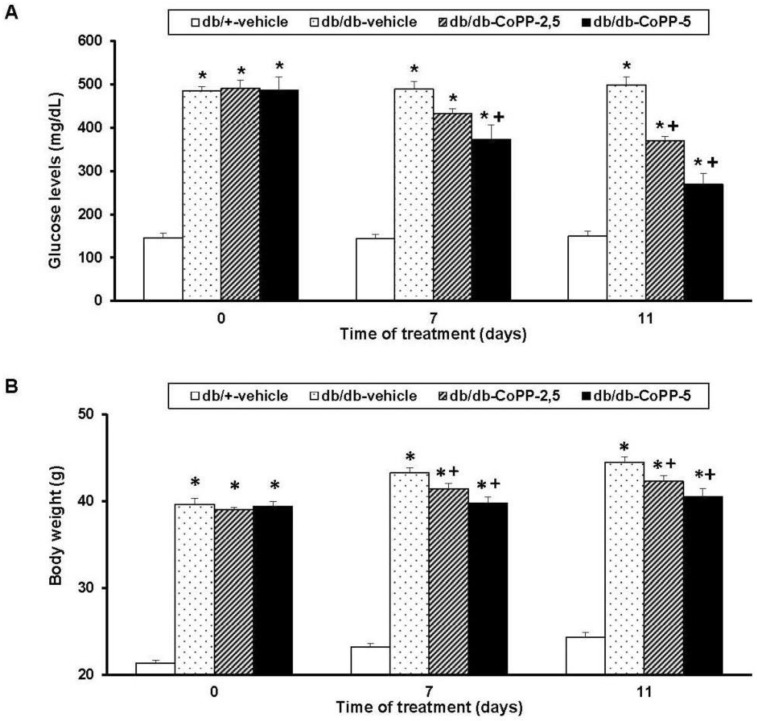
Effects of CoPP treatment on blood glucose levels and body weight in db/db mice. The glucose levels (mg/dL) and body weight (g) in db/db mice treated with vehicle or CoPP at 2.5 and 5 mg/kg during 0, 7 and 11 consecutive days are shown. Glucose levels (**A**) and body weight (**B**) in db/+ mice treated with vehicle are also represented. For each parameter and day evaluated, * indicates significant differences as compared to db/+ mice treated with vehicle and + indicates significant differences as compared to db/db mice treated with vehicle (*p <* 0.05 one-way ANOVA followed by the Student Newman Keuls test). Each column represents the mean and vertical bars indicate SEM; *n* = 6 animals for each group.

On days 0, 7 and 11 of treatment with 2.5 and 5 mg/kg of CoPP, glucose levels (Figure 2A) and body weight (Figure 2B) were measured. For glucose levels, a significance effect of the treatment (*p* < 0.001), time of administration (*p* < 0.001) and its interaction (*p* < 0.001) was revealed by a two way ANOVA repeated measures. Therefore, the high blood glucose levels proved in db/db mice were diminished by CoPP administered at 5 mg/kg after 7 and 11 days of treatment (*p* < 0.001; one-way ANOVA vs. vehicle db/db treated mice) as well as in animals treated with CoPP at 2.5 mg/kg during 11 days (*p* < 0.001; one-way ANOVA vs. vehicle db/db treated mice).

Regarding body weight, an effect of treatment (*p* < 0.001) and time (*p* < 0.001) but not their interaction was demonstrated. Certainly, the increased body weight in db/db mice was significantly inhibited by the treatment with 5 or 2.5 mg/kg of CoPP during 7 and 11 days, respectively (*p* < 0.001; one-way ANOVA vs. vehicle db/db treated mice).

### 2.4. Effects of CoPP on Nrf2, HO-1, NQO1 and JNK Expression in Sciatic Nerve

**Figure 3:**
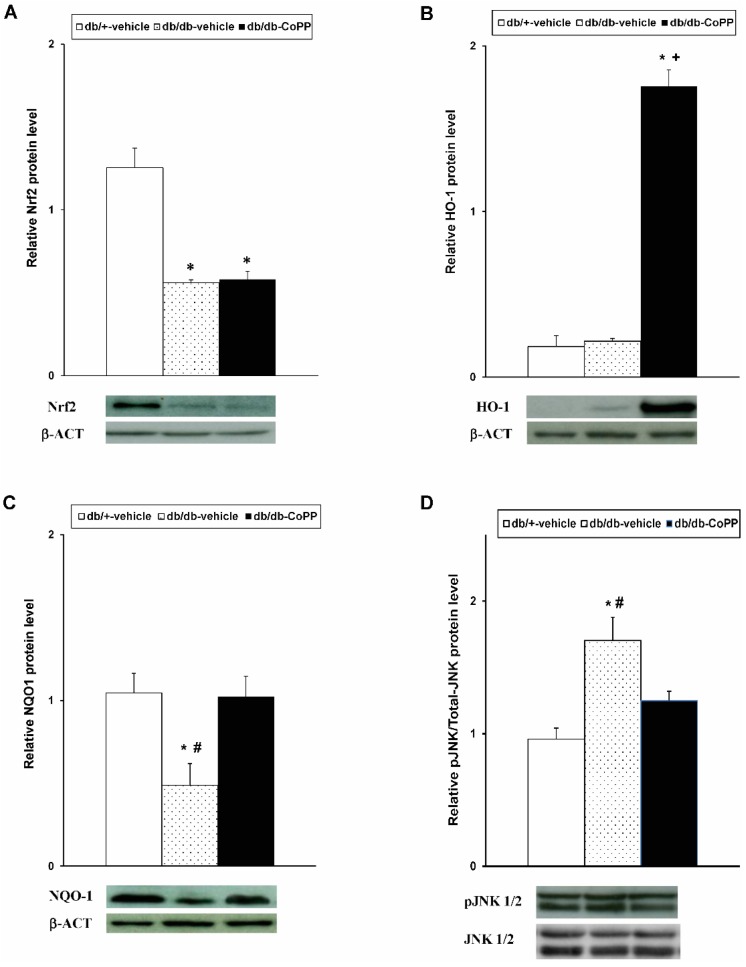
Effects of CoPP treatment on the protein levels of Nrf2, HO-1, NQO1 and JNK in the sciatic nerve of diabetic mice. The protein levels of Nrf2 (**A**), HO-1 (**B**), NQO1 (**C**) and JNK (**D**) in the sciatic nerve from db/db mice treated with CoPP or vehicle and in db/+ mice treated with vehicle are represented. For each protein, * indicates significant differences as compared to db/+ mice treated with vehicle and # indicates significant differences vs. db/db mice treated with CoPP (*p* < 0.05, one-way ANOVA, Student Newman Keuls test). Examples of western blots for Nrf2, HO-1, NQO1 and pJNK proteins in which β-actin or total JNK were used as loading controls are also shown. Each column represents the mean and vertical bars indicate SEM; *n* = 4 samples per group.

Results indicated that while the expression of Nrf2 was significantly reduced in the sciatic nerve of db/db mice (Figure 3A, *p* < 0.001; one-way ANOVA vs. db/+ vehicle treated mice), the expression of HO-1 (Figure 3B) did not change in db/db mice. Moreover, whereas CoPP treatment did not modify the Nrf2 levels, an up-regulation of HO-1 was demonstrated in CoPP treated diabetic mice (*p* < 0.004; one-way ANOVA vs. db/+ and db/db vehicle treated mice). Our findings also showed that the diminished expression of NQO1 in the sciatic nerve of db/db mice was reversed by CoPP treatment (Figure 3C; *p* < 0.017; one-way ANOVA vs. db/db mice treated with vehicle). Concerning JNK, the increased phosphorylation detected in the sciatic nerve of db/db mice (Figure 3D; *p* < 0.001; one-way ANOVA) was inhibited with CoPP treatment.

### 2.5. Effects of JWH-015 and JWH-133 on the Mechanical Allodynia

**Figure 4:**
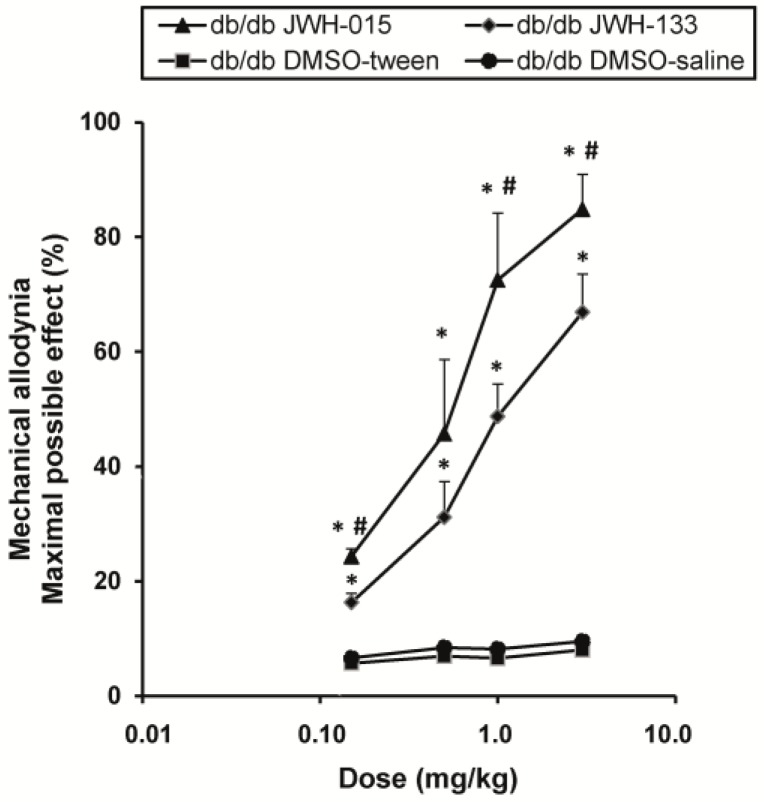
Antiallodynic effects of JWH-015 or JWH-133 in db/db mice. Antiallodynic effects of the subcutaneous administration of different doses (logarithmic axis) of JWH-015 or JWH-133 and their respective controls in the hind paws of db/db mice are represented. Each point represents the mean value of maximal possible effect (%) and vertical bars indicate SEM (6 animals for each dose). For each drug and dose, * indicates significant differences vs. their respective db/db vehicle treated mice and # indicates significant differences vs. effects produced by JWH-133 (*p <* 0.05, one-way ANOVA followed by the Student Newman Keuls test).

The administration of JWH-015 or JWH-133 (Figure 4) inhibited allodynia in db/db mice in a dose-dependent manner. Thus, the antiallodynic effects of 0.15, 0.5, 1 and 3 mg/kg of JWH-015 or JWH-133 were significantly higher than their corresponding vehicles (*p <* 0.002, one way ANOVA and Student Newman Keuls test). Moreover, the antiallodynic effects of JWH-015 administered at 0.15, 1 and 3 mg/kg were also higher than those produced by JWH-133 (*p <* 0.01, one way ANOVA and Student Newman Keuls test).

The intraperitoneal administration of AM630, a selective CB2R antagonist, at 1 mg/kg completely reversed the effects displayed by 3 mg/kg of JWH-015 or JWH-133 in db/db mice (*p <* 0.001; one way ANOVA and Student Newman Keuls test; Table 1) and did not have any effect on db/db vehicle treated mice.

### 2.6. Effects of CoPP and SFN Treatments on the Antiallodynic Effects of JWH-015 and JWH-133

**Figure 5:**
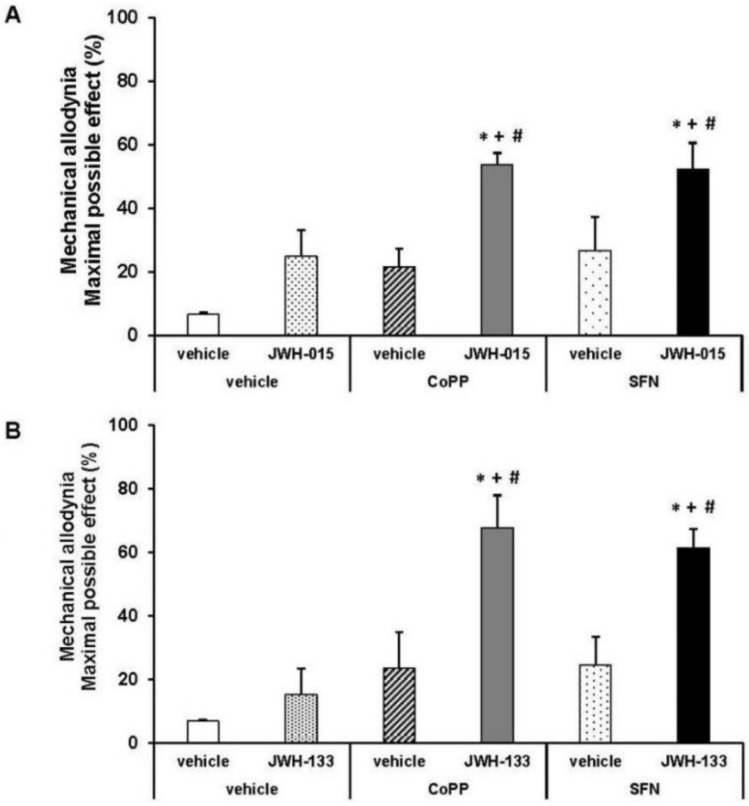
Effects of the co-administration of CoPP or SFN with JWH-015 or JWH-133 on the inhibition of mechanical allodynia in diabetic mice. Antiallodynic effects of the subcutaneous administration of 0.15 mg/kg of JWH-015 (**A**) or JWH-133 (**B**) or their respective vehicle in the hind paws of db/db mice pretreated with 5 mg/kg of CoPP, 10 mg/kg of SFN or vehicle are represented. The effects of CoPP and SFN intraperitoneally administered alone are also shown. For each drug tested, * denotes significant differences vs. mice treated with vehicle plus vehicle or vehicle, + denotes significant differences vs. animals treated with vehicle plus JWH-015 or JWH-133 and # denotes significant differences vs. animals treated with CoPP or SFN plus vehicle (*p <* 0.05; one way ANOVA followed by the Student Newman Keuls test). Each column represents the mean value of maximal possible effect (%) and vertical bars indicate SEM; *n* = 6 animals for each group.

Figure 5 showed the antiallodynic effects of 0.15 mg/kg of JWH-015 (A) or JWH-133 (B) subcutaneously administered and their respective vehicles in db/db mice pre-treated with 5 mg/kg of CoPP, 10 mg/kg of SFN or vehicle (DMSO 1%) by via intraperitoneal. CoPP and SFN treatments augmented the inhibitory effects of JWH-015 (A) or JWH-133 (B) in comparison to their respective groups treated with vehicle plus vehicle or drug, and to animals receiving CoPP or SFN plus vehicle (*p <* 0.01, one way ANOVA and Student Newman Keuls test).

### 2.7. Effects of CoPP and SFN Treatments on CB2R Levels in the Dorsal Root Ganglia of db/db Mice

**Figure 6:**
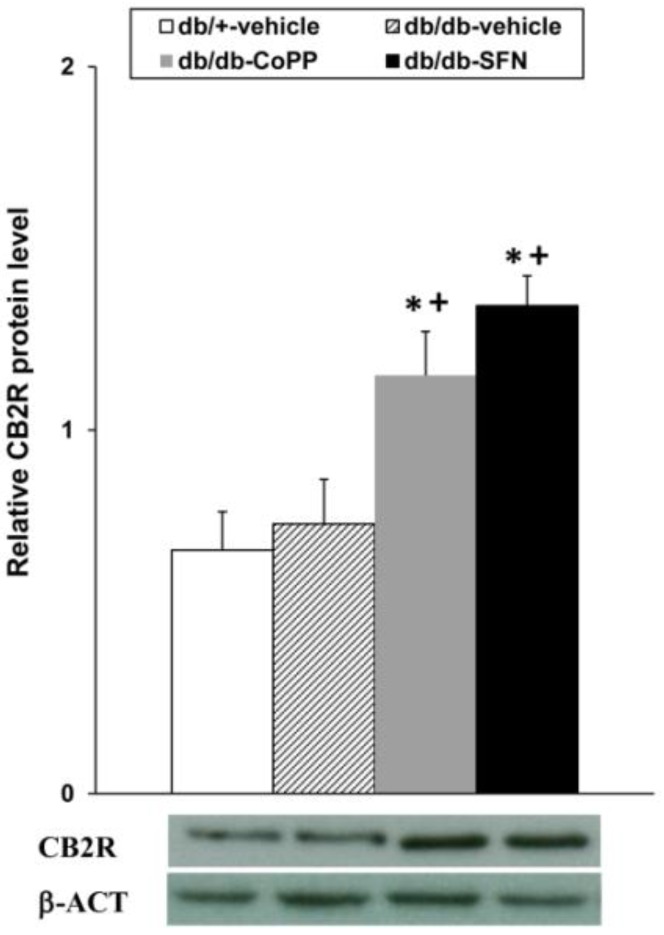
Effects of CoPP and SFN treatments on the protein levels of CB2R in the dorsal root ganglia from diabetic mice. Protein levels of CB2R from db/db mice treated with CoPP, SFN or vehicle are represented. The expression of CB2R from db/+ mice treated with vehicle has been also shown as controls. * indicates significant differences when compared vs. db/+ mice treated with vehicle and + indicates significant differences when compared vs. db/db mice treated with vehicle (*p <* 0.05, one-way ANOVA followed by Student Newman Keuls test). A representative example of western blot for CB2R, in which β-actin was used as a loading control, is also shown. Each column represents the mean value and vertical bars indicate SEM; *n* = 4 samples per group.

Our results demonstrated that both CoPP and SFN treatments increased the CB2R levels in the dorsal root ganglia from db/db mice compared to db/db and db/+ vehicle treated mice (Figure 6; *p <* 0.001; one-way ANOVA, Student Newman Keuls test).

## 3. Discussion

Treatment with CoPP blocked allodynia and inhibited the increased glucose levels and body weight gain manifested in db/db mice. CoPP besides to enhance HO-1 expression also regularized the down regulation of NQO1 and avoided JNK activation in sciatic nerve of diabetic mice. Moreover, both CoPP and SFN treatments improved the antiallodynic effects of CB2R and its expression in the dorsal root ganglia of db/db mice.

Our and other groups demonstrated the relevant anti-inflammatory and antinociceptive actions produced by the induction of HO-1 in several animal pain models [8,26,27] as well as in type 1 diabetic animals [11,13]. This study validated these data and furthermore demonstrated that CoPP inhibited allodynia in db/db mice revealing their antinociceptive efficiency in type 2 diabetes. In accordance to that, the stimulation of the Nrf2 transcription factor, principal inductor of HO-1 synthesis, also inhibited neuropathy in type 1 and 2 diabetic animals [13,20], showing that the Nrf2/HO-1 signaling pathway activation plays a key role alleviating the nociceptive behaviors linked to diabetes.

Our data also showed that the enlarged body weight in db/db mice was prevented by CoPP treatment demonstrating the anti-obesity properties of HO-1 in these animals. In accordance to our results, the stimulation of HO-1 and Nrf2 inhibited body weight gain in high-fat-diet-induced obesity and in obese ob/ob rodents [28,29,30]. A possible mechanism by what HO-1 protects from obesity might be by degrading the pro-oxidant heme which produces potent anti-oxidant substances, for example carbon monoxide and bilirubin, both capable to inhibit obesity [5].

It is well known that HO-1 might increase glucose tolerance in type 2 diabetic animals by blocking the synthesis of inflammatory mediators [31,32]. Our results supported these data showing that CoPP treatment significantly reversed high blood glucose levels in db/db mice by inducing the antiinflammatory/antioxidant enzyme, HO-1. In accordance to us, augmented levels of HO-1 have been also proven in type 1 diabetic animals treated with many HO-1 inductors [11,33,34] and in type 2 diabetic mice treated with a transcription factor Nrf2 activator [20]. In accordance to the potent antinociceptive properties of CoPP under inflammatory and neuropathic pain conditions [7,8,11], the HO-1 up-regulation induced by CoPP might be also the main responsible for the inhibition of allodynia induced by this compound in our type 2 diabetic model. Nonetheless, the significant reduction of glucose levels induced by CoPP might also contribute to its analgesic actions in db/db mice.

NQO1 is other enzyme also involucrate in the protection against oxidative stress [35], as demonstrated by the increased reactive oxygen species and hyperglycemia produced by the disruption of this gene in mice [12]. Therefore, as predictable, a diminished expression of NQO1 was established in the sciatic nerve of db/db mice [13,20] which was normalized by CoPP treatment indicating that the antiallodynic actions of CoPP might be also facilitated by the reestablishment of NQO1 protective effects in db/db mice. Curiously, the fact that CoPP treatment rescued the NQO1 levels in the sciatic nerve of db/db mice, but not those of Nrf2, suggested that CoPP can induce NQO1 expression in a non Nrf2-expression dependent manner. However, possible changes in the Nrf2 transcription factor activity induced by CoPP treatment cannot be discarded. In addition, the inhibition of JNK phosphorylation induced by CoPP might also indicate that NQO1 expression is induced by CoPP via activating JNK pathway. Nevertheless, more experiments are needed to confirm this hypothesis.

MAPK (JNK, ERK and p38) are also implicated in the modulation of diabetic neuropathy [14,36], so activated MAPK were demonstrated in the dorsal root ganglia and/or sciatic nerve of db/db mice and its inhibition attenuated pain sensitivity displayed in these animals at the early phase of diabetes [15,16,20]. In conformity to that, we demonstrated an increased phosphorylation of JNK in the sciatic nerve of db/db mice which was reversed by CoPP, as occurs with this treatment in insulin resistant type 2 diabetic rats [37], as well as by SFN (a NRF2 activator) in db/db mice [20], revealing e that the antiallodynic effects triggered by the Nrf2/HO-1 signaling pathway in animals with type 2 diabetes were in part mediated via JNK inhibition.

It is amply recognized that cannabinoids are markedly implicated in the modulation of neuropathic pain, such as caused by nerve or spinal cord injury as well as those related to diabetes [21,38,39]. Thus, the administration of selective cannabinoid 1 receptors (CB1R) or CB2R and non-selective CB1R/CB2R agonists (WIN 55,212-2) inhibited painful neuropathy in animals with type 1 or 2 diabetes [21,22,23,40]. The present investigation demonstrated that two specific CB2R agonists (JWH-015 and JWH-133) were also capable to inhibit the mechanical allodynia induced by type 2 diabetes in a dose-dependent manner. In addition, a significant interaction between CB2R and the Nrf2/HO-1 signaling pathway was demonstrated with the enhancement on the antiallodynic properties and expression of CB2R in diabetic mice pre-treated with Nrf2 or HO-1 inducers. Therefore, the administration of SFN or CoPP augmented the protein levels of CB2R in the dorsal root ganglia of db/db mice and in consequence the inhibitory effects of JWH-015 or JWH-133 in CoPP or SFN pre-treated db/db mice. It is interesting to mention that in contrast to the important side effects related to the activation of CB1R, less undesirable effects are induced by CB2R activation [41]. Therefore, the demonstration of the antiallodynic actions of two specific CB2R agonists (JWH-015 or JWH-133) in diabetic mice and the improvement of its effects with CoPP or SFN resulting an interesting approach for treating diabetic neuropathy avoiding the CB1R agonists associated site effects. In accordance to our data, a significant interaction between the Nrf2/HO-1 pathway and the opioid and cannabinoid systems has been also demonstrated in diabetic animals. Indeed, while the activation of Nrf2 improved the analgesic effects of DOR in db/db mice [20], carbon monoxide generated by HO-1 also potentiated the inhibitory actions displayed by DOR and CB2R agonists in type 1 diabetic mice [21,42].

In conclusion, CoPP treatment inhibited mechanical allodynia, reduced glucose levels and body weight gain in db/db mice by enhancing HO-1/NQO1 levels and reducing JNK phosphorylation. Both CoPP and SFN treatments improved the antiallodynic properties of JWH-015 and JWH-133, and the expression of CB2R in db/db mice. These findings revealed that stimulation of antioxidant Nrf2/HO-1 way potentiated the effects of CB2R agonists and might be suitable for treating painful neuropathy linked to type 2 diabetes.

## 4. Material and Methods

### 4.1. Animals

We employed 8-week old male type 2 diabetic mice (BKS.Cg-m+/+Leprdb/J; db/db) and heterozygotes as controls (db/+) acquired from Charles River Laboratories (France). Mice were accommodated under 12-h/12-h light/dark conditions in a room with controlled temperature of 22 °C and humidity of 66%. Animals with free access to food and water were used after 6 days acclimatization to housing conditions. All experiments were conducted between 9:00 and 17:00 and executed according to the animals guidelines of the European Communities Council (86/609/ECC, 90/679/ECC; 98/81/CEE, 2003/65/EC, and Commission Recommendation 2007/526/EC) and approved by the Comissió d’Etica en l’Experimentació Animal i Humana de la Universitat Autònoma de Barcelona (number: 6266). Maximal exertions to reduce suffering and number of animals employed were made.

### 4.2. Nociceptive Behavioral Test

*Mechanical allodynia* was measured by evaluating the hind paw withdrawal response to von Frey filament stimulation, a similar method has been published before [11,20]. Briefly, mice were sited in methacrylate cylinders (20 cm-high, 9 cm-diameter) with a wire grid bottom across which the von Frey filaments (North Coast Medical, Inc., San Jose, CA, USA) with a bending force in the range of 0.008–3.5 g were applied by using an adapted version of up–down paradigm [43]. We start the test with the filament of 0.4 g and the filament of 3.0 g was utilized as a cut-off. The strength of next filament was decreased or increased depending on the answer, and threshold of response was calculated from sequence of filament strength used throughout the up–down procedure using an Excel program (Microsoft Iberia SRL, Barcelona, Spain) which contains curve fitting of the data. Clear paw withdrawal, licking or shaking the paw was considered to be a nociceptive-like response. To obtain appropriate behavioral immobility, animals were habituate for 1 h before testing. Both hind paws were tested.

### 4.3. Western Blot Analysis

Control and db/db mice injected during 11 days with vehicle, 5 mg/kg of CoPP or 10 mg/kg of SFN were sacrificed by cervical dislocation, 3 h after the last administration of CoPP or SFN. Tissues from sciatic nerve and dorsal root ganglia (L3 to L5) were extracted, frozen in liquid nitrogen and maintained at −80 °C. Samples from two animals were pooled together to assure adequate protein levels for doing western blot assay to analyze Nrf2, HO-1, NQO1, total JNK, phosphorylated JNK and CB2R protein levels.

Tissues were homogenized in ice-cold lysis buffer (50 mM Tris·Base, 150 nM NaCl, 1% NP-40, 2 mM EDTA, 1 mM phenylmethylsulfonyl fluoride, 0.5 Triton X-100, 0.1% sodium dodecyl sulfate, 1 mM Na3VO4, 25 mM NaF, 0.5 % protease inhibitor cocktail, and 1% phosphatase inhibitor cocktail). All reagents were acquired from Sigma (St. Louis, MO, USA), except NP-40 from Calbiochem (Darmstadt, Germany). Homogenate was solubilized 1 h at 4 °C, sonicated 10 s and centrifuged 15 min at 4 °C and 700 g. Supernatant (60 μg of total protein) was mixed with laemmli loading buffer (4×) and loaded onto 4% stacking/10% separating sodium dodecyl sulfate polyacrylamide gels. Proteins were electrophoretically transferred onto PVDF membrane for 120 min, blocked with PBS or TBST + 5% nonfat dry milk or TBST + 5% BSA and then incubated at 4 °C overnight with a rabbit anti-Nrf2 (1:160, Abcam, Cambridge, UK), anti-HO-1 (1:300, Abcam, Cambridge, UK), anti-NQO1 (1:350, Sigma, St. Louis, MO, USA), anti-phosphorylated JNK (1:250, Cell Signaling Technology (Danvers, MA, USA), anti-total JNK (1:250, Cell Signaling Technology (Danvers, MA, USA) and anti-CB2R (1:500, Abcam, Cambridge, UK) antibodies. Proteins were detected by a horseradish peroxidase-conjugated anti-rabbit secondary antibody (GE Healthcare, Little Chalfont, Buckinghamshire, UK), visualized with chemiluminescence reagents (ECL kit; GE Healthcare, Little Chalfont, Buckinghamshire, UK) and by exposure onto hyperfilm (GE Healthcare). Blots intensity was quantified by densitometry. Membranes were stripped and reproved with a monoclonal rabbit anti-β-actin antibody (1:5000, Abcam, Cambridge, UK) used as a loading control. For JNK, data are expressed as the ratio of density of phosphorylated JNK to total JNK.

### 4.4. Experimental Procedure

For mechanical allodynia, after habituation for 1 h to von Frey filaments test during 4 days, baseline measurements were obtained. Body weight and glucose levels from one drop of tail blood were assessed with a glucometer (OneTouch® UltraMini®, Lifescan, Johnson & Johnson, NJ, USA) (*n* = 6 mice for each group, in total 12 animals).

The effects on mechanical allodynia, glucose levels and body weight induced by CoPP intraperitoneally injected at 2.5 and 5 mg/kg were evaluated (*n* = 6 mice for each dose). We used db/+ mice administered with vehicle as controls (*n* = 6 mice for each group). For mechanical allodynia, animals were evaluated before and at 1, 3, 5, 7, 9 and 11 days of treatment with CoPP or vehicle. Body weight and blood glucose levels were evaluated before and at 7 and 11 days of treatment with CoPP or vehicle. We used in total 24 animals.

We also assessed the effects of the subcutaneous administration of 0.15, 0,5, 1 and 3 mg/kg of two CB2R agonists (JWH-015 and JWH-133) on the mechanical allodynia in db/db mice (*n* = 6 mice per group, in total 96 animals). Reversion of the antiallodynic effects of 3 mg/kg of JWH-015 and JWH-133 were evaluated with a CB2R antagonist (AM630) intraperitoneally administered at 1mg/kg (*n* = 6 mice per group, in total 36 animals).

In other experiments, the antiallodynic effects of pre-treatment with 5 or 10 mg/kg of CoPP or SFN in animals treated with 0.15 mg/kg of JWH-015 or JWH-133 were also studied (*n* = 6 mice per group, in total 72 animals). In these experiments CoPP and SFN were intraperitoneally administered 3 h before CB2R agonists injection. The doses of CoPP and CB2R agonists were determined from dose-responses curves conducted in this work and that of SFN was selected in accordance to a preceding study [20].

At 11 days of treatment with CoPP (5 mg/kg), SFN (10 mg/kg) or vehicle, db/db mice were sacrificed and tissues extracted for evaluating the protein levels in sciatic nerves and dorsal root ganglia by western blot. We used vehicle db/+ treated mice as controls (*n* = 4 samples for each group, 32 animals). In total we used 272 animals.

### 4.5. Drugs

CoPP obtained from Frontier scientific (Livchem GmbH Co, Frankfurt, Germany) and SFN of Merck Chemicals and Life Sciences S.A.U. (Madrid, Spain) were dissolved in dimethyl sulfoxide (DMSO; 1% solution in saline) and administered at 3 h before behavioral testing. Doses of both compounds were chosen from preceding pilot studies and other works [8,11,20]. JWH-015, JWH-133 and AM630 were acquired from Tocris (Ellisville, MI, USA). JWH-015 and AM630 were dissolved in DMSO and JWH-133 was dissolved in DMSO and Tween 80. All drugs were subcutaneously administered 30 min before testing. Doses of both compounds were chosen from preceding pilot studies and other works [8,11,20]. All drugs were prepared immediately before use and injected in a final volume of 10 mL/kg. In all groups, control animals received their respective vehicle.

### 4.6. Statistical Analysis

The Statistical Package for Social Sciences (SPSS, version 17 for Windows, IBM (Madrid, Spain)) was utilized to perform statistical analysis. Data are shown as mean ± standard error of the mean (SEM). The evaluation of mechanical allodynia, glucose levels and body weight between db/db and db/+ mice was performed with an unpaired Student’s *t*-test. The effects of continuous administration of different doses of CoPP on mechanical allodynia, glucose levels and body weight in db/db mice in comparison to vehicle treated db/+ mice were analyzed with a two-way ANOVA for repeated measures (treatment and time as between factors of variation), followed by a one-way ANOVA and Student Newman Keuls test.

The antiallodynic effects of different doses of JWH-015, JWH-133 or vehicle were evaluated by using a one way ANOVA and the Student Newman Keuls test. The reversal of JWH-015 or JWH-133 effects with AM630 was also analyzed with a one way ANOVA and Student Newman Keuls test. The comparison of the effects of CoPP and SFN on the antiallodynic actions of JWH-015 or JWH-133 was also assessed by a one way ANOVA and Student Newman Keuls test. The antinociception in von Frey filaments was expressed as the percentage of maximal possible effect, where the test latencies pre (baseline) and post-drug administration were compared and calculated according to the next equation:

maximal possible effect (%) = [(drug − baseline)/(cut-off − baseline)] × 100(1)

Changes in protein levels of Nrf2, HO-1, NQO1, JNK and CB2R in sciatic nerve or dorsal root ganglia of db/db mice treated with CoPP or SFN and vehicle vs. vehicle db/+ treated mice were evaluated by a one-way ANOVA and Student Newman Keuls test. A value of *p <* 0.05 was considered significant.

## Figures and Tables

**Table 1:** Reversion of the antiallodynic effects of JWH-015 and JWH-133 (3 mg/kg; subcutaneously administered) by the intraperitoneal administration of 1 mg/kg of AM630 in db/db mice.

Treatments	von Frey Filaments Strength (g)		
vehicle + vehicle	1.7	±	0.1
JWH-015 + vehicle	2.8	±	0.1 *
JWH-133 + vehicle	2.6	±	0.1 *
vehicle + AM630	1.6	±	0.1
JWH-015 + AM630	1.7	±	0.1
JWH-133 + AM630	1.7	±	0.2

Results are shown as mean values ± SEM; *n* = 6 animals per experimental group. * indicates significant differences vs. db/db mice treated with vehicle plus vehicle (*p* < 0.05, one-way ANOVA, Student Newman Keuls test).
